# Skin nerve phosphorylated α-synuclein in the elderly

**DOI:** 10.1093/jnen/nlae015

**Published:** 2024-02-26

**Authors:** Vincenzo Donadio, Laura Fadda, Alex Incensi, Alessandro Furia, Sara Parisini, Francesco Colaci, Giovanni Defazio, Rocco Liguori

**Affiliations:** IRCCS Istituto delle Scienze Neurologiche di Bologna, UOC Clinica Neurologica, Bologna, Italy; Azienda Ospedaliero Universitaria di Cagliari, SC Neurologia, Cagliari, Italy; IRCCS Istituto delle Scienze Neurologiche di Bologna, UOC Clinica Neurologica, Bologna, Italy; IRCCS Istituto delle Scienze Neurologiche di Bologna, UOC Clinica Neurologica, Bologna, Italy; IRCCS Istituto delle Scienze Neurologiche di Bologna, UOC Clinica Neurologica, Bologna, Italy; IRCCS Istituto delle Scienze Neurologiche di Bologna, UOC Clinica Neurologica, Bologna, Italy; Department of Biomedicine and Translational Neuroscience, Aldo Moro University of Bari, Bari, Italy; IRCCS Istituto delle Scienze Neurologiche di Bologna, UOC Clinica Neurologica, Bologna, Italy; Dipartimento di Scienze Biomediche e Neuromotorie, Università di Bologna, Bologna, Italy

**Keywords:** α-Synuclein misfolded, Centenarians, Elderly subjects, Skin biopsy, Specificity

## Abstract

To determine the incidence of phosphorylated α-synuclein (p-syn) in skin nerves in very old subjects who are prone to developing incidental Lewy bodies, we prospectively performed skin biopsies on 33 elderly subjects, including 13 (>85 years old) and 20 patients (>70 years) suspected of having an acquired small fiber neuropathy. All subjects underwent neurological examination prior to the biopsy. Two screened female subjects (ages 102 and 98 years) were excluded from the study because they showed evidence of a slight bradykinetic-rigid extrapyramidal disorder on neurological examination and were not considered healthy; both showed p-syn in skin nerves. We did not identify p-syn in skin nerves in the remaining 31 subjects. A PubMed analysis of publications from 2013 to 2023 disclosed 490 healthy subjects tested for skin p-syn; one study reported p-syn in 4 healthy subjects, but the remaining subjects tested negative. Our data underscore the virtual absence of p-syn in skin nerves of healthy controls, including those who are very elderly. These data support skin biopsy as a highly specific tool for identifying an underlying synucleinopathy in patients in vivo.

## INTRODUCTION

Reliable and easily accessible biomarkers for Parkinson disease (PD) and other synucleinopathies are still lacking. This prevents the identification of these disorders in vivo and assessment of the effects of disease-modifying treatments. We have reported specific tests that identify pathological and misfolded α-synuclein (α-syn) in the skin of live patients (1, 2). Skin biopsy with immunofluorescence (IF) staining for phosphorylated (misfolded) α-synuclein (p-syn) in nerves is among the most promising diagnostic tools for identifying specific PD biomarkers (3–8). According to a reported meta-analysis, IF staining has very good diagnostic accuracy in identifying abnormal α-syn deposits in PD in vivo (9). The reliability of IF as a diagnostic tool has also been supported by excellent inter- and intra-laboratory reproducibility (10).

Although skin p-syn shows excellent sensitivity and specificity (3–8, 11–13), a potential problem of using this biomarker for PD and other synucleinopathies is the presence of incidental Lewy bodies (ILB) in elderly subjects, as found in the brain (14–19), and in peripheral nerves (20, 21) of autopsy cases. The incidence of p-syn deposits in the skin of very elderly subjects has not been determined to date.

The aim of this study was to ascertain the in vivo presence of skin p-syn in very elderly subjects who were not affected by a synucleinopathy based on clinical examination. We also reviewed published reports to clarify the overall occurrence of ILB in healthy subjects. This information will help establish the feasibility of identifying abnormal aggregation of skin p-syn for differentiating healthy individuals from those with an underlying synucleinopathy. The data will help to define the reliability of this biomarker for PD and other synucleinopathies.

## MATERIALS AND METHODS

### Subjects

We prospectively screened and performed skin biopsies on 33 consecutive elderly subjects including 13 very elderly healthy (>85 years) and 20 patients (>70 years) suspected of having an acquired small fiber neuropathy because of burning feet and predisposing reasons for a peripheral neuropathy (Table 1).

**Table 1.** Demographic data of recruited subjects who underwent skin biopsy

Subjects	Number	Sex M:F	Age (years), mean±SD	Symptom duration (years)
Healthy	11*	5:6	93 ± 7	NA
Pt with possible SFN	20	9:11	79 ± 4	3 ± 2
All subjects	31*	14:17	84 ± 4	3 ± 2

F, female; M, male; NA, not applicable; Pt, patients; SFN, small fiber neuropathy.

* Two elderly subjects aged 102 and 98 years were excluded from the healthy group because they showed parkinsonian signs on neurological examination.

All subjects were evaluated by a neurologist with expertise in movement disorders (D.V. or F.L.) to exclude any possible motor and nonmotor signs that might indicate the presence of a synucleinopathy. They underwent a neurological examination and specific clinical scales and questionnaires for cognitive (MoCA), autonomic (COMPASS-31), and motor (UPDRS-III and Hoehn and Yahr [H&Y]) functions. The procedures used were approved by the local Human Ethics Committee and followed the Helsinki Declaration regarding international clinical research involving human beings. All subjects gave their written informed consent to the study.

### Skin biopsy

Three-millimeter punch biopsies were taken from proximal cervical C7 paravertebral area in all subjects, as this is the site with the highest rate of p-syn positivity in Lewy body diseases (10, 11, 13, 22–24). Furthermore, the skin biopsy was also performed in distal hairy skin sites including thigh (15 cm above the patella) and distal leg (10 cm above the lateral malleolus). The distal skin biopsy was performed on only 2 healthy subjects for the thigh and 4 healthy subjects for the leg. The remaining subjects declined to undergo the extensive skin biopsy protocol, which was considered too demanding for their ages. As previously reported, in each skin site we take a second biopsy 3–4 cm away from the first sample to increase the rate of detection of p-syn positivity (10, 11, 13, 22–24). Skin samples were immediately fixed in cold Zamboni fixative and kept at 4° C overnight, according to previously published procedures (22–24).

Ten-micrometer sections were obtained using a freezing sliding microtome (CM 1950; Leica, Deerfield, IL). Sections were double-immunostained overnight with a panel of primary antibodies including rabbit monoclonal phosphorylated α-synuclein at Ser 129 ([p-syn]; 1:500, Abcam, Cambridge, United Kingdom, cat. no. ab-51253) and mouse pan-neuronal marker protein gene product 9.5 ([PGP]; 1:750; Abcam, Cambridge, United Kingdom, cat. no. ab72911). Sections were then washed and secondary antibodies were added for an incubation of 1 hour. As secondary antibodies, an anti-mouse Alexa Fluor(R) 488 (1:400; Jackson ImmunoResearch, West Grove, PA, cat. num. 715-545-150) and rabbit cyanine dye fluorophores 3.18 (1:200, Jackson ImmunoResearch; cat. num. 711-165-152) were used. The microscope analysis and criteria followed to define a p-syn positivity were previously described (1, 5, 11, 13, 14). Shortly, sections were initially viewed and analyzed under a Zeiss fluorescent microscope. The correspondence between rabbit p-syn and mouse PGP staining helped to verify the intraneuronal deposits excluding possible nonspecific staining arising from the background. The analysis was made in a blinded fashion to the clinical diagnosis by 2 authors with expertise in immunofluorescence analysis (D.V. and I.A.). P-syn staining was rated as positive when a single skin nerve fiber showed a positive staining at high magnification (×40). The colocalization of p-syn with PGP was analyzed in digital images acquired by a laser-scanning confocal microscope (Nikon confocal microscopy, Eclipse Ti A1, Tokyo, Japan). Each image was collected in successive frames of 1- to 2-μm increments on a Z-stack plan at the appropriate wavelengths for secondary antibodies with a ×20 or ×40 plan apochromat objective and subsequently projected to obtain a double-stained digital image by an imaging analysis software (NIS, Nikon Imaging Software, Eclipse Ti A1).

### Review analysis: search strategy and eligibility criteria

For the searching analysis, we used PubMed. The search string was “skin biopsy” AND “phosphorylated alpha-synuclein” or “skin” AND “alpha-synuclein.” The search was not filtered for study type. Two authors independently assessed studies and any disagreement was resolved by consensus with a third author. Original data were thus revised. Criteria for study selection and consideration were original studies, case reports, case-controls design, use of p-syn in skin nerves and the report of healthy subjects. Reviews and autopsy studies were not considered. We thus assessed 18 studies from the period 2013–2023. Ten papers were not considered because different forms of α-synuclein (i.e. synuclein total or native, oligomer form) were analyzed.

## RESULTS

### Clinical screening of recruited subjects

**Figure 1.**
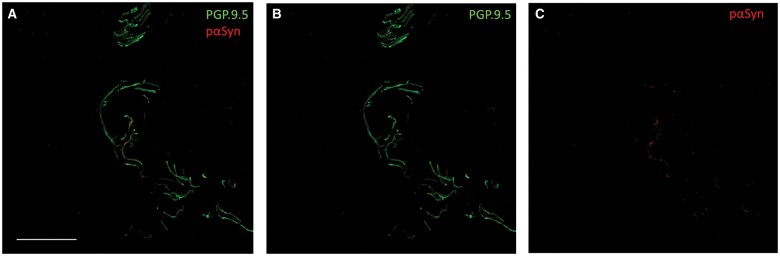
Immunofluorescence staining of phosphorylated α-synuclein (p-syn) in skin nerves in a screened subject. **(A–C)** Confocal microscope (×400) study of p-syn in skin nerves of a 103-year-old female subject who was screened for this study. Because the neurological examination demonstrated a mild bradykinesia with slight rigidity predominantly in the right hemisome with a UPDRS-III score of 23 and H&Y of 1, she was not considered as a “healthy subject.” Abnormal p-syn deposits were found in nerves of a plexus in the deep dermis. P-syn was demonstrated by anti-Ser 129 phosphorylated P-syn **(C)**; the neuronal plexus was identified using anti-PGP 9.5 **(B)**. Abnormal p-syn deposits were in neuritic inclusions, as shown in the merged image (**A**). These abnormal synuclein deposits support the diagnosis of synucleinopathy in this subject.

Among the 13 healthy subjects screened (8 females; mean age of 94 years, ranging from 86 to 103 years), 2 female subjects aged 102 and 98 years were excluded from the study because they showed parkinsonian signs on neurological examination that were not reported during the clinical interview. They showed a modest bradykinesia with a slight rigidity prevalently in the right or left hemisome (UPDRS-III of 23 and 25, respectively and H&Y of 1 for both). Although they were not included in the study of healthy subjects, skin biopsy was nevertheless carried out at the C7 site to establish the possibility of identifying p-syn deposits in a very early phase of PD. Abnormal p-syn aggregates were found in skin nerves in both patients (Fig. 1), supporting the possibility of identifying these abnormal aggregates in the early disease phase of PD. This also emphasizes the correlation between the presence of these aggregates with clinical signs of PD. Both patients declined to allow additional examinations to better characterize the skin biopsy findings (i.e. brain MRI, nigrostriatal DatScan) due to the mild clinical disability and the difficulty of returning to the hospital because of their ages.

There were 11 healthy subjects recruited for this study (6 females, mean age of 92 years, range from 86 to 103 years), without symptoms of synucleinopathy. Patients who needed a skin biopsy because of a suspect of an acquired small fiber neuropathy (10 females, mean age of 79 years, from 71 to 88 years) were recruited because of their ages and absent symptoms of synucleinopathy (i.e. cognitive, parkinsonian, cerebellar or autonomic dysfunctions). Predisposing causes for peripheral neuropathy included diabetes (8 patients), autoimmune connective diseases (8), hepatitis C (2), and deficiency of B12 vitamin (2). In all recruited subjects the neurological examination and MoCA, COMPASS-31, and UPDRS-III and H&Y were in the ranges of normality, thereby excluding possible signs associated with a synucleinopathy.

### Skin biopsy

Abnormal aggregates of p-syn in skin nerves were not found in any healthy subjects and patients. A small fiber neuropathy was found in 18 out of 20 patients showing predisposing causes for peripheral neuropathy (Table 1).

### Review analysis

A total of 490 healthy subjects were analyzed using p-syn staining with a neuronal marker. In these studies, abnormal p-syn deposits were reported in a single paper in 4 subjects of unspecified age (Table 2).

**Table 2.** Studies selected for the review analysis

Authors	(Reference)	Skin biopsy sites	Number of healthy subjects investigated	Rate of p-syn positivity (%)	Mean age
Donadio et al, 2013	(13)	C8, T, L	15	0	62 ± 11 (SD)
Doppler et al, 2014	(14)	T12, T, L	35	0	59.9 (35–80)
Donadio et al, 2014	(25)	C7, T, L	30	0	67 ± 7 (SD)
Doppler et al, 2015	(5)	T12, L	39	0	61
Antelmi et al, 2017	(7)	C7, T, L	55	0	NR
Donadio et al, 2017	(22)	C7, T, L	25	0	67 ± 12 (SD)
Donadio et al, 2018	(13)	C7, T, L	10	0	70 ± 3 (SE)
Melli et al, 2018	(8)	C8, T, L	12	0	57
Kuzkina et al, 2019	(26)	C7, T10, T, L	21	0	58 ± 12 (SD)
Carmona-Abellan et al, 2019	(27)	C, T, L	2	0	NR
Al-Qassabi et al, 2021	(28)	C8	21	0	60.8 ± 8.2 (SD)
Wang et al, 2020	(2)	T, L	21	0	62.6 ± 10.7 (SD)
Liu et al, 2020	(29)	C7, Fo, T, L	30	0	59.10 ± 10.93
Vacchi et al, 2021	(30)	C8, L	22	18	60 ± 11
Nolano et al, 2022	(31)	C8, Fi, L	30	0	NR
Giannoccaro et al, 2022	(32)	C7, T, L	26	0	67 ± 10.9 (SD)
Donadio et al, 2023	(33)	C7, T, L	50	0	65 ± 1 (SE)
Gibbons et al, 2023	(12)	C7, T, L	24	0	62 ± 8 (SD)

C7, cervical C7 spine; C8, cervical C8 spine; T10, thoracic T10 spine; T12, thoracic T12 spine; C, cervical; T, thigh; Fo, forearm; Fi, finger; L, leg; NR, not reported; SE, standard error; SD, standard deviation.

## DISCUSSION

Our study showed: (1) absent abnormal aggregates of p-syn in skin nerves of very elderly subjects without signs of synucleinopathy; (2) abnormal skin p-syn in 2 elderly subjects who did not complain of symptoms but showed signs of a slight bradykinesia on neurological examination; and (3) virtually absent skin p-syn deposits in a large number of healthy subjects reported in the recent scientific literature.

These data underscore the virtual absence of p-syn in the skin innervation of controls, including healthy individuals, even those who are elderly, such as centenarians. These findings support the reliability of skin biopsy as a biomarker for PD and other synucleinopathies, as it is a highly specific tool to identify patients with an underlying synucleinopathy in vivo.

ILB refer to the presence of Lewy bodies and/or Lewy neurites in the CNS and PNS of elderly individuals not known to be affected by parkinsonism and/or dementia (14–21). The incidence of abnormal α-synuclein aggregates in the brain increases with age; it has been reported as 4.7% in the sixth decade, 9.3% in the seventh decade, and 12.8% in the eighth decade (34, 35). In an autopsy case study of the elderly African population, the prevalence of ILB was 5.3% (36). However, the prevalence data of ILB in postmortem series are highly variable, ranging from 0% (37) to 34% (38). Taken together, the reported mean prevalence of ILB is approximately 10%–15% of the autopsy population (14–21, 33–37). In addition, isolated p-syn deposits in the peripheral autonomic nerves in the heart and adrenal gland without evidence of CNS Lewy bodies have also been reported in autopsies (38–40). This suggests the possibility of ILB restricted to the PNS as a prodromal phase of pure autonomic failure.

We reviewed the literature to identify healthy subjects analyzed using the immunofluorescence technique with the aim of detecting p-syn in skin nerves.. They had an age comparable to that of the autopsy series investigating ILB in the general population (14–21, 33–37). Considering the expected prevalence rate of 10%–15% in the autopsy studies and the possibility of disclosing ILB in the PNS, we expected that ∼50 subjects should display ILB and a p-syn positivity in skin nerves. However, we did not find p-syn positivity in any of the recruited subjects. The review analysis revealed that only 4 healthy subjects reported in one study (41), out of 490 subjects investigated, were positive for p-syn. These data emphasize that ILB are virtually absent in the skin and the peripheral nerve endings, suggesting that ILB could differ from pathological conditions represented by synucleinopathies characterized by frequent deposition of α-synuclein in skin nerves. The contrasting finding in comparison to autopsy data could be explained by a lack of seeding pathological activity of misfolded α-synuclein underlying ILB differently from the abnormal aggregates underlying diseases characterizing synucleinopathies. This conclusion is supported by previous autopsy studies disclosing ILB mainly in peripheral nerves (20, 21), but not in nerve endings of the skin (39). However, a different study reports these aggregates sporadically in the skin of ILB cases (42). Alternatively, our findings can be explained by considering that reported autopsy cases were usually defined on the basis of the clinical history alone, without neurological evaluation. Therefore, it cannot be excluded that the reported cases of ILB may not actually have shown minimal parkinsonian or autonomic signs not evidenced by the clinical histories. In fact, 2 of our healthy elderly cases did not report any neurological symptoms in their clinical history but neurological examination disclosed mild extrapyramidal dysfunction, as a possible expression of a synucleinopathy demonstrated by the presence of p-syn deposits in skin nerves. Our cases highlight how subtle clinical signs of synucleinopathy may be overlooked in patient medical histories. We report these cases in this article as an internal control to emphasize the correlation of abnormal α-synuclein deposits in skin nerves with the early preclinical PD stage in centenarian patients in contrast to their absence in healthy subjects of similar ages.

This study has 2 important novel findings: first, the search for p-syn in the skin nerves of very elderly (included centenarian) subjects has not previously been reported. This is evident in Table 2 that reports the data from recent previous papers in which the average age of the healthy controls analyzed ranged from 57 to 70 years; the second novel finding is that for the first time we reviewed literature to establish the p-syn positivity in skin nerves using an immunofluorescence technique in a large cohort of healthy subjects. This analysis supports the virtual absence of p-syn deposits in skin nerves and supports the data we obtained in our healthy subjects, indicating that they may extend to a larger group of healthy subjects. In fact, by cumulatively considering the reviewed and the original data of the very elderly and centenarian subjects reported in this work, the established specificity of this technique in differentiating healthy controls from patients suffering from a synucleinopathy is very high (99%). This study supports the use in clinical practice of skin biopsy and the search of skin p-syn by immunofluorescence as a highly specific tool to identify a pathological condition related to synucleinopathies.

Our study has the following limitations. First, the lack of an autopsy study in our recruited subjects prevents us from demonstrating ILB in the brain or peripheral nerves. Thus, we cannot exclude that our recruited subjects had ILB in other parts of the body. However, this aspect is irrelevant for the objective of this study, which was to evaluate ILB in skin nerves in very elderly subjects to support the specificity of skin biopsy as a tool for synucleinopathy. In future studies, the evaluation of the relationship between the correspondence of p-syn deposits in brain, peripheral nerves, and skin could be an important goal to clarify the seeding activity of misfolded α-synuclein in the incidental form of synucleinopathies, and to better understand the pathological characteristics of misfolded α-synuclein in ILB. The second limitation of this study is the small sample size of analyzed subjects, mainly due to the difficulty in recruiting very elderly subjects for even a minimally invasive test. Our conclusions are also supported by the reported findings of the virtual absence of p-syn deposits in hundreds of healthy subjects.
